# Retraction: DJ-1 Modulates α-Synuclein Aggregation State in a Cellular Model of Oxidative Stress: Relevance for Parkinson's Disease and Involvement of HSP70

**DOI:** 10.1371/journal.pone.0219023

**Published:** 2020-01-24

**Authors:** The PLOS ONE Editors

Concerns have been raised about a number of western blot bands presented in the figures of this article [1]. Specifically:

- Fig 1C: bands 1–3, 4 and 5 of the lower panel are similar in appearance to the bands in lanes 5–3, 2 and 1, respectively (flipped horizontally) in the α-tubulin blot in Fig 5A of [2]. Bands 1–3 of the lower panel are similar in appearance to the bands in lanes 5–3 (flipped horizontally) of the β-actin panel in Fig 4B of [3]. The bands in lanes 4 and 5 of the lower panel are similar in appearance to the bands in lanes 1 and 2 (flipped vertically) of the β-actin panel in Fig 4B of the same article [3].
- Fig 2A: bands in lanes 2, 3 and 4 of the lower panel have a similar appearance to each other.
- Fig 2C: bands in lanes 2, 4, and 7 have a similar appearance to each other; bands in lanes 1, 3, and 5 have a similar appearance to each other.
- Fig 4C: in the DJ-1 and HSP70 panels, bands in lanes 1 and 2 have a similar appearance to each other; bands in lanes 5 and 6 have a similar appearance to each other; in the HSP70 panel bands in lanes 3 and 4 have a similar appearance to each other.
- Fig 4E: the western blot panels appear similar to those published in Fig 6C of [3].
- Fig 5A: bands in lanes 7–9 of the bottom TAT-α-syn panel have a similar appearance to each other; in the α-tubulin panel, bands in lanes 3–4 have a similar appearance to bands in lanes 5–6; bands in lanes 1 and 2 of the α-tubulin panel have a similar appearance to each other; in the DJ-1 panel, bands in lanes 1–3 have a similar appearance to bands in lanes 4–6.

The original uncropped image files for the western blot panels listed above are no longer available. The corresponding author acknowledged the removal of molecular weight standard sample lanes and splicing together of non-adjacent lanes in the preparation of the figures, causing the appearance of vertical lines in the background of the images. The uncropped blot image for the upper panel of Fig 2A was provided, showing that lane 4 of the original blot contained the molecular weight marker, which was spliced out of the image used in Fig 2.

The corresponding author has stated there was an error in the selection of blot images used for Fig 4E and has provided an uncropped image of the correct blot.

As part of the underlying dataset for the study, an uncropped blot generated during the set up of aggregation experiments was provided. Lanes 1–5 in the uncropped blot are similar in appearance to lanes 1–5 of Fig 3D, while lanes 6–8 appear different. Labelling of this uncropped blot is not consistent with labelling of Fig 3D.

The *PLOS ONE* Editors have been unable to verify the accuracy and reliability of the western blot data presented in the paper. In light of the extent of the unavailability of the underlying original western blot data and the unresolved concerns regarding the accuracy and reliability of the figures, the *PLOS ONE* Editors retract this article.

As discussed above, Fig 4E and the α-tubulin panel of Fig 1C are similar to or duplicate content published in [3], published 2004 *The FASEB Journal*, which is not offered under a CC-BY license. Permissions were not obtained at the time of the article’s [1] original publication to use and offer these images under the CC-BY license. *PLOS ONE* has obtained permissions from FASEB to retain these images in the retracted article under a non-CC-BY license. Fig 4E and the α-tubulin panel of Fig 1C are excluded from this article’s [1] CC-BY license. At the time of retraction, the article [1] was republished to update the Copyright Statement and add information about the original source and reproduction permissions for these images to the legends for Figs 1 and 4. Please see the complete, correct Fig 1 and Fig 4 legends here:

**Fig 1. TAT-α-syn and TAT-DJ-1 prevent oxidative stress in SK-N-BE cells.** (A) Dose-response pattern of the toxic effect of hydrogen peroxide (H_2_O_2_) in SK-N-BE cells. (B) Dose-response curve of 6-hydroxydopamine (6-OHDA) toxicity in SK-N-BE cells. Cells were plated and challenged by oxidative stress for 24 h and cell viability was assessed by erythrosine-dye exclusion assay; *p<0.05; ***p<0.001 vs control group (0 μM H_2_O_2_ or 6-OHDA), Dunnett’s *post-hoc* test. (C) Western blot assessing TAT-α-syn 0.5 μM and TAT-DJ-1 3 μM availability inside SK-N-BE cells. Cells were plated and incubated in presence of TAT-α-syn or TAT-DJ-1 for the reported time intervals. To demonstrate equal gel loading, α-tubulin immunoreactivity is also presented. (D) Protective effect of TAT-delivered α-syn and (E) TAT-delivered DJ-1 against oxidative stress. Cells were incubated with increasing amounts of TAT-α-syn or TAT-DJ-1 2–4 h before the toxic treatment and 24 h later cell viability was assessed by erythrosine-dye exclusion assay; *p<0.05; **p<0.01 vs control group at the same TAT-α-syn or TAT-DJ-1 concentration (CT, corresponding also to 0 μM H_2_O_2_ or 6-OHDA), Tukey’s *post-hoc* test. (F) Toxicity of micromolar amounts of TAT-α-syn. Cells were incubated with increasing concentrations of TAT-α-syn for 24 h and cell viability was assessed by erythrosine-dye exclusion assay. **p<0.01 vs control group (CT, corresponding to 0 μM TAT-α-syn), Dunnett’s *post-hoc* test. (G) Amyloid aggregation of TAT-α-syn 3 μM was detected by thioflavin-T staining after 24 h incubation; a) SK-N-BE control cells; b) SK-N-BE cells incubated with TAT-α-syn 3 μM. The arrows indicate intracellular thioflavin-T-positive inclusions (magnification 20X). Owing to concerns about similarities with previously published content in [36], the α-tubulin panel of Fig 1C is excluded from this *PLOS ONE* article’s CC-BY license; FASEB granted permissions to retain the image in this retracted article. See the accompanying retraction notice for more information.

**Fig 4. TAT-DJ-1 affects HSP70 mRNA expression.** (A) Digital image of a capillary electrophoresis assessing the effect of DJ-1 on HSP70 mRNA expression. Cells were either incubated with TAT-DJ-1 for 24 h or silenced for DJ-1 expression for 72 h. Then total mRNA was extracted, reverse-transcribed and amplified to detect HSP70 (550 bp band) and aldolase-A (180 bp band), used as internal control. The bar graph (B) shows the RT-PCR assay values and is representative of one of three independent experiments (*n* = 4 for each group). CT-: siRNA negative control; **p<0.01 vs control (siRNA lipid vehicle alone), Tukey’s *post-hoc* test. (C) Western blot showing HSP70 protein expression. Cells were incubated with either TAT-DJ-1 for 24h or DJ-1 siRNA for 72 h, then harvested for Western blotting. The bar graph (D) shows the calculated values normalized to α-tubulin as internal control and is representative of one of three independent experiments (*n* = 3 for each group); ***p<0.001 vs control group (siRNA lipid vehicle alone), Tukey’s *post-hoc* test. (E) Western blotting assessing HSP70 expression level in SK-N-BE cells incubated with negative control TAT-fused proteins. Cells were incubated with TAT-GFP or TAT-α-syn(1-97) for 24 h and then harvested to perform HSP70 immunodetection. The western blot panels in Fig 4E report material from [36], reproduced with permission of FASEB. The images in Fig 4E are excluded from this *PLOS ONE* article’s CC-BY license. See the accompanying retraction notice for more information.

DA, AN, GF agreed with the retraction. RR, SB, LP, FP, MP either did not respond or could not be reached.
